# Network analysis of marmoset cortical connections reveals pFC and sensory clusters

**DOI:** 10.3389/fnana.2024.1403170

**Published:** 2024-06-12

**Authors:** Bernard A. Pailthorpe

**Affiliations:** Brain Dynamics Group, School of Physics, The University of Sydney, Sydney, NSW, Australia

**Keywords:** marmoset, connectivity, network, module, cluster, hub, PFC

## Abstract

A new analysis is presented of the retrograde tracer measurements of connections between anatomical areas of the marmoset cortex. The original normalisation of raw data yields the fractional link weight measure, FLNe. That is re-examined to consider other possible measures that reveal the underlying in link weights. Predictions arising from both are used to examine network modules and hubs. With inclusion of the in weights the InfoMap algorithm identifies eight structural modules in marmoset cortex. In and out hubs and major connector nodes are identified using module assignment and participation coefficients. Time evolving network tracing around the major hubs reveals medium sized clusters in pFC, temporal, auditory and visual areas; the most tightly coupled and significant of which is in the pFC. A complementary viewpoint is provided by examining the highest traffic links in the cortical network, and reveals parallel sensory flows to pFC and via association areas to frontal areas.

## 1 Introduction

Our early knowledge of brains came from a variety of classic neuroanatomical methods, that now include tracing neural pathways. Modern network science (Bullmore and Sporns, 2009; Bassett and Sporns, 2017) offers one model of connectivity between biologically significant areas of the cortex. Computational studies of such models can reveal local structures worthy of further study. The marmoset brain has well developed frontal lobes, so provides an accessible system for studying cortical function. In evolutionary terms marmoset is well advanced beyond mouse, yet simpler than higher primates, so provides a good entry point for developing simple models of cortical function. The Rosa lab has accumulated significant data from tracer injections and labelled cell counts to provide a measure of link weights in the marmoset cortex (Majka et al., 2016, 2020). That study sampled 55 injection sites chosen amongst 116 anatomical areas. They also conducted a detailed network analysis of the fully connected, or edge-complete, sub-network of those 55 areas (Theodoni et al., 2022) using the original fractional linkage weight measure. That found a hierarchical ordering from frontal areas continuing through, motor, somatosensory and association areas with visual areas at the lowest level. In a separate analysis of the 55 area sub-network Liu et al. (2020) conducted a comprehensive network analysis including finding the community, or modular, structure of the network and identifying hub nodes. They (Liu et al., 2020) identified frontal, auditory and association areas as most hub like, followed by visual areas, and singled out A10 as having unusually strong out links.

The present study is an analysis of the partially sampled network of all 116 areas of the marmoset cortex using a re-normalisation of the original data that reveals the individual in-links weights. Thus it extends the earlier analyses by including more data. While still incomplete, it does include valuable additional data that reveals the underlying in-link weights and includes out-links to non-sampled areas. Given that the choice of the 55 injection sites was guided by expert biological insights, the available data is likely to capture a significant fraction of the important links in the cortex. It is noteworthy that the data and analysis described above, and used herein, refer to parcellation via anatomical areas by neuroanatomists (Paxinos et al., 2012). Other, biologically relevant, approaches include the structural model (García-Cabezas et al., 2019), that uses a smaller class of cortical types, or categories of cortical areas with similar laminar differentiation and connection strengths. Predictions based on this model are starting to emerge (Aparicio-Rodríguez and García-Cabezas, 2023), with comparisons of link weight fall off with both physical link length vs. cortical type distance. This model offers a complimentary biological viewpoint from which to conduct network analyses. To my knowledge, further network analysis on the basis of the structural model is yet to be undertaken.

There is also an opportunity to apply a suite of network methods, used previously for worm, mouse retina, mouse, rat and macaque to further study marmoset cortex. Various groups have used different measures of connectivity, including link weights, depending on the experimental techniques used (EM, tracer, MRI) and the research questions examined. Thus synaptic contact areas (mouse retina), tracer volume and raw labelled cell counts (mouse brain) and fractional measures (macaque and marmoset cortex) have been used. Consistency with prior research is one reason for a given choice, while the merits of the varying approaches are still being assessed. The analysis methods used herein follow those developed and tested on the mouse retina (Pailthorpe, 2016) and the mouse brain (Rubinov et al., 2015; Pailthorpe, 2019), and are compared with other choices.

The marmoset network data is at mesoscale, describing links between anatomical areas described in the Paxinos atlas (Paxinos et al., 2012). Variability of repeated tracer injection volumes suggested Fraction of Labelled Neurons, extrinsic, FLNe as a measure of link strength, derived from retrograde tracer data from macaque cortex (Markov et al., 2011, 2014), and as originally reported for the marmoset data used herein. The present study uses the more extensive, recent marmoset data (Majka et al., 2020) to re-normalise that fraction to a more direct measure of source-target link weights based on LNe, the number of Labelled Neurons, extrinsic. This is consistent with the mouse brain tracer data (Oh et al., 2014) that reported links weights as raw connection strength, CS (Rubinov et al., 2015), specifically the measured volume of fluorescent tracer detected in a linked target area. That should be proportional to the number of labelled neurons—essentially equivalent to LNe in the present notation—a question examined herein. The benefit of this approach is that it discriminates varying in-link weights to target nodes, and then incorporates those weights in the subsequent analysis.

It is illustrative to begin the network analysis with counts of in- and out-links to/from nodes, i.e., the node Degree, followed by summing weights of in- and out-links to find the weighted in- and out-Degree of each node (also called the node strength in some of the network literature). While elementary, revisiting those measures reveals consistencies within the dataset and with other studies, and suggests the new approach adopted herein. The distributions are variously exponential, normal or log normal, depending on the species, methods used, and scale of the measured areas. The present analysis of the marmoset data also covers: dependencies of the basic link weight measurements on injection and target volumes that facilitates a re-analysis of the raw data using a rescaled measure of links weights (cf. Methods, Supplementary material); link weight distribution; link weight-distance plots to confirm exponential decay; modular decomposition using InfoMap and Louvain methods, and identification of network hubs; and tracing evolving links around hub nodes to identify local clusters and pathways in the cortex, along with sensory pathways. Link tracing around key hubs reveals local clusters in pFC, auditory, association and visual areas; with the pFC cluster being the most tightly interlinked. Visualisation of pathways around hubs and connectors, and those hosting high network traffic, suggest parallel pathways from auditory and visual sensory areas to pFC and motor areas. By contrast modular decomposition based on the fractional measure FLNe, using both InfoMap, herein (cf. Supplementary material 2.6), and Louvain methods (Theodoni et al., 2022), gives prominence to the visual areas. That analysis revealed link placement driven by spatial embedding, possibly consistent with the structural model; and also a laminar dependence of links. Separately (Liu et al., 2020) conducted a comprehensive network analysis of the same 55 node sub-network, and identifies frontal and auditory cortices as prominent modules, consistent with the present analysis.

## 2 Materials and methods

Mesoscale connectivity data for the marmoset cortex is available as a link list text file (Majka et al., 2016)^1^ from retrograde tracer injections to 55 target areas selected from amongst 116 anatomical areas of the cortex. This data is for ipsi-lateral links in the left hemisphere. Characteristics of the original experiments have been reported in detail (Majka et al., 2020; Theodoni et al., 2022). In summary, there were 3474 links from a possible 116 source areas; that is 26% of the possible links in the subsampled full network. This is significantly sparser than the 62.4% fraction of links in the full connected 55 x 55 sub-network previously analysed (Theodoni et al., 2022). Use of more injection sites likely will yield a larger fraction of links amongst the 166 nodes, intermediate between those two values. A summary of technical terms used herein is included in Table 1.

**TABLE 1:** Summary of technical terms.

Adjacency matrix—a mathematical array of link weights between source and target nodes of a network; equivalent to a connectivity matrix;
Area—anatomical area delineated via parcellation defined using expert anatomical methods;
Connector node—has many links to other modules;
Degree (k)—a count of the number of in or out links to/from a network node;
FLNe—a fractional measure of link weight (defined in Methods);
Hub—a key node with many links between modules, or with many links preferentially within a module;
Infomap—a random walk based algorithm to decompose a network into component modules—it optimises a code length describing that decomposition;
Link—a connection between nodes in a network (e.g.,. fibre tract, road, rail routes, etc.);
LNe–a measure of link weight (defined in Methods);
Module–a sub network of nodes that are more strongly linked within the group than to those outside; also called community.
Network—a collection of nodes and links that approximately models a real-world system;
Node—a point in a network that acts as a source or target of links between nodes; used here in a network model to represent an anatomical area, etc;
Participation coefficients–a measure of the fraction of a node’s links that are within a module, compared to all its links;
Probability flow–a measure of the fraction of random walks that pass through a given node; it can also be calculated for links.;
Strength, or weighted node Degree—the sum of all link weights to/from a network node;
Weighted Degree—weighted node degree: the sum of link weights in or out of a node; also called strength;
z score–the within module weighted degree, for in or out links, measured in standard deviations from the mean.

### 2.1 Measured distribution of links and weights, FLNe

**FIGURE 1:**
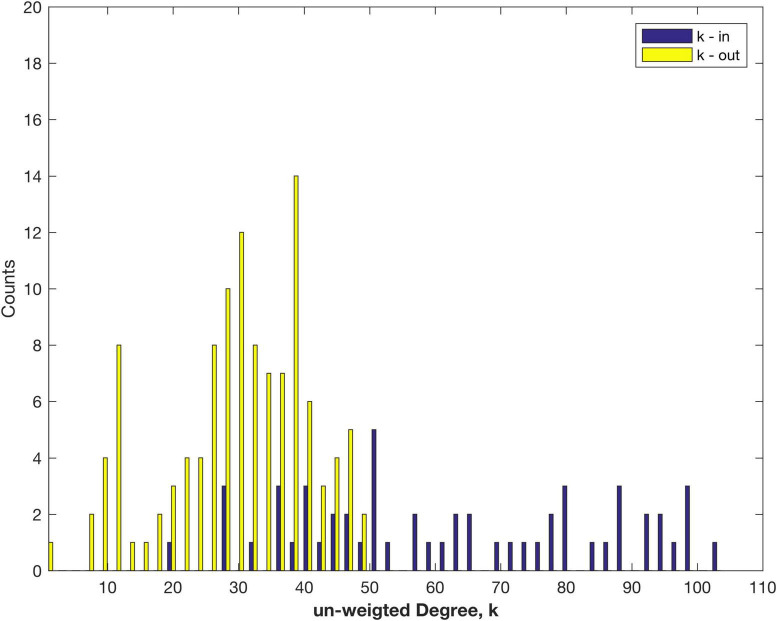
Distribution of the number of in- and out-links (un-weighted Degree), k measured for areas of the marmoset cortex.

The measured link in-weights were normalised to form the Fraction of Labelled Neurons extrinsic, FLNe, a measure that was used for earlier macaque data (Markov et al., 2011, 2014). Thus for each node all the fractional in-weights sum to 1, so that differences between nodes are factored out, and the weighted in-Degree of each connected node is 1, or 0 if no links were measured. However, this data does preserve the number of links. This is reflected in the plot of in-Degree, k-in, as a distribution of the number of in-links, presented in Figure 1, along with k-out for the out -inks.

The maximum number of out links is 48, much less than the maximum, 104, of in links. The clear asymmetry arises from only 55 target nodes (i.e., tracer injection sites) being measured thus far, from amongst 116 possible sources. There are likely to be some other targets not yet captured in the present dataset. The number of out links is better fit by a normal, rather than log normal, distribution (log likelihood comparison), as discussed below. For the in links the distribution is better fit by an exponential distribution—that may change as more target sites are studied and more data becomes available (note the smaller number of counts). The data set still shows the variation in the number of in-links to each node (Figure 1). Note that the analysis of the fully connected, 55 node sub-network produces more symmetry between in- and out-Degree, since that sub-sampling ensures that there are no missing links: cf. Figure 2B of Theodoni et al. (2022).

**FIGURE 2:**
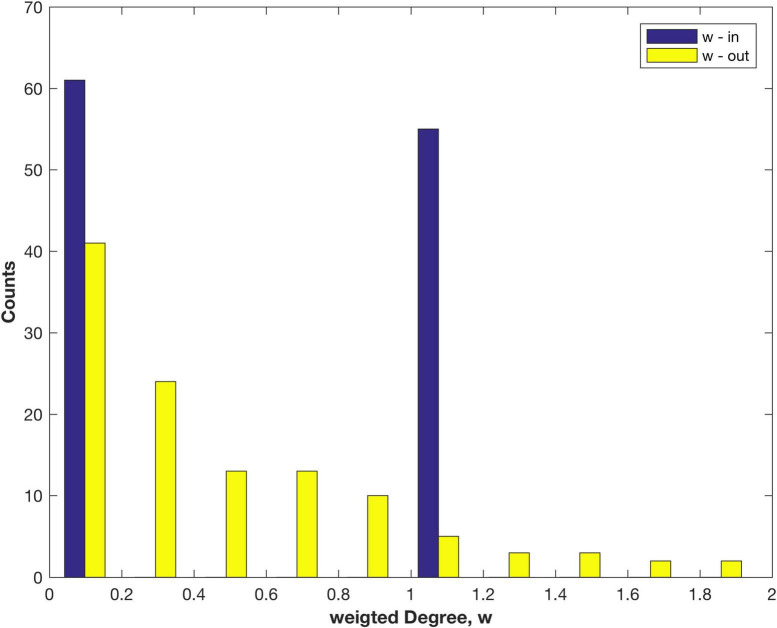
Distribution of the weighted in- and out-Degree (node strength) measured as FLNe for areas of the marmoset cortex.

The weighted Degree, also called the node strength, distributions found using FLNe are shown in Figure 2. The out-Degree appears to have an exponential decay, as observed for other species, e.g., the nematode worm C. Elegans (Varshney et al., 2011), mouse retina (Pailthorpe, 2016) and mouse brain (Oh et al., 2014; Pailthorpe, 2019). However, the original weighted in-Degree adopts only values of 0 or 1 since fractional measures sum to 1 for each node, which follows from the definition of FLNe. That result is a tell-tale sign, pointing to an opportunity for further analysis. It arises since FLNe factors out differences of in-link weights between nodes, so that some network information is discarded. The combined distribution of all FLNe weights is shown in Supplementary Figure 1.

Note that a similar analysis of the 55 x 55 connectivity matrix would produce incomplete results since it is formed by deleting the 61 empty columns belonging to the non-injection sites. It also requires deletion of the corresponding 61 rows that do contain data, being 1611 non-zero links: these are the out links from the non-sampled areas to any of the 55 injection sites. That step excluded 26% of the total link weight measured in the cortex, which now is included in the present study. Those out links (A-B) also happen to be in links (B-A) that were counted in forming FLNe. Those are sampled in Figure 2.

### 2.2 Rescaled link weight data, LNe

In this analysis the original FLNe data is rescaled to be consistent with the mouse data (Oh et al., 2014) and to uncover the underlying in-link weights. This is possible with the more recent, larger set of measurements (Majka et al., 2020) that includes sufficient detail to enable the fractional normalisation, used above, to be recalculated. Here a cortex wide, common normalisation for all nodes (representing anatomical areas) is adopted so that the underlying weights of in links can be recovered. The full data is described in detail in Supplementary material 2.2 and the procedure used to calculate LNe is detailed in Supplementary material 2.3. The new data uses the same 55 target nodes as the original data, multiple tracers, with repeated experiments, so 143 experimental results are available. Extensive raw experimental data was reported: i.e., each injection volume, number of Labelled Neurons (LN) both intrinsic (to the injected volume) and extrinsic (meaning all source neurons), and their total: LNi, LNe, LNtot, respectively. This extra data enables the normalisation, that was used to calculate FLNe, to be recovered and to produce the underlying link weights. That normalisation factor varies for each target node since it is a measure of the weighted in Degree, or node strength. Dependencies of LNi and LNe on injection volumes and node volumes are explored in Supplementary Figures 4, 5, and analysed in Supplementary material 2.4, and informs the choice of link weight measure.

Here the direct measure, LNe, is used so that the underlying weight distribution for the in-links is revealed. LNe is the number of labelled neurons, extrinsic (i.e., not in the target area, and including all those detected in related source areas). This is consistent with the mouse brain tracer data (Oh et al., 2014) that reported links weights as raw connection strength (CS), specifically the measured volume of fluorescent tracer in a linked target area. That should be proportional to the number of labelled neurons—essentially equivalent to LNe in the present notation. A caveat for that comparison is that it assumes uniform and constant neuronal density: that has been measured for Marmoset (Atapour et al., 2019) and exhibits ∼10% variation between some areas, and an Anterior-Posterior gradient.

In network terms the measured LNe is equal to the weighted in-Degree of the injected target node: this can be calculated both for out links (sum over linked targets) and for in links (sum over linked sources). In an explicit notation, the reported FLNe(s, t) is the fractional link weight from source s to target t. Similarly LNe(t) identifies the injection site for the retrograde tracer with the target node. This LNe(t) is the total weight of incoming links from linked source nodes: i.e., LNe(t) = Σ LNe(s, t), where the sum is over all linked source nodes (s). That sum is just the weighted in-Degree of the target node (cf. Results 2.1). It varies across nodes (i.e., areas) while, by contrast, the corresponding fractional measure FLNe(t) is either 0 (no incoming links), or 1 (target linked from one or more sources); cf. Figure 2. The array of LNe(s, t) is just the network adjacency matrix, attached at Supplementary Data, which forms the basis of subsequent calculations. The detailed procedure for its calculation is presented in Supplementary material 2.3.

### 2.3 Network modules and hubs

Here the InfoMap method was used to decompose the network into modules, that are sub-collections of nodes that have more linkages between nodes within a module than between them. Results are reported using both data sets, based on FLNe and on LNe. The InfoMap method and software^2^ models random walks over the network (Rosval and Bergstrom, 2008), and thus provides insight into how information flows over the network. The method optimizes the modular dcomposition by maximising the mutual information of each assignment of nodes and links into a specified number of modules, amongst all possible combinations. This is equivalent to finding a minimum length code that describes the modular decomposition [cf. Figure 1 of (Rosval and Bergstrom, 2007)]. The optimisation metric in this method is the so-called Huffman code length [explained in Figures 1, 2 of (Rosval and Bergstrom, 2008)], for which a minimum is sought. The InfoMap algorithm is preferred since it is conceptually based on network traffic, i.e., multiple random walkers traversing all possible paths on the network; and has been shown to yield good results in a variety of applications (Fortunato, 2010). Limitations of these methods has been studied on a large collection of networks (Ghasemian et al., 2019) and reveals that InfoMap tends to over-fit the data in that it provides good link description but poor link prediction. For comparison, a simple implementation of the Louvain method (Blondel et al., 2008)^3^ with default settings was also used. It agglomerates nodes and then re-iterates to optimise a modularity metric, Q based on counting links (Newman and Girvan, 2004), finding *Q* = 0.52, which is a mid-range value. This Q measures the fraction of links within modules, compared to random linkages. These two methods generally produce similar results despite being conceptually quite different algorithms.

A related analysis, originally applied to metabolic networks within a cell (Guimerà and Nunes Amaral, 2005), leads to identification of network hubs. Two measures are calculated and plotted together: the participation coefficient of node i, P_i_, is a measure of the fraction of a node’s links within a module, compared to all its links. The limit P_i_→1 indicates that the node has wide ranging links between modules, while P_i_→0 indicates that the nodes links are primarily local, within its own modules. The second measure, the z-score, z_i_ indicates how well connected a node is to other nodes in its own module (a membership score, or within-module Degree measured in standard deviations from the mean). These measures can be calculated separately for in-links and out-links. 2D plots of z_i_ vs. P_i_ have been divided into regions that classify nodes and types of hubs, as illustrated in the figures below. The regions in the 2D plot and the hubs’ classifications follow the heuristics developed for metabolic networks (Guimerà and Nunes Amaral, 2005) to describe classes or roles assigned to network nodes. The important ones are: Role 6 (R6), connector hubs (many links between modules); R5, provincial hubs (links preferentially within module); R4, non-hub kinless (links across most, or all, modules); and R3, non-hub connectors (many links to other modules). A related analysis has been applied previously to cat and macaque brain networks (Sporns et al., 2007). That study used a binary connection matrix, i.e., unweighted links. For comparison the module decomposition computed using marmoset FLNe is presented in Supplementary Table 2 and discussed in the Supplementary material. The difference in in-weights in the two methods produced different module decompositions.

The key measure that separates hubs from non-hubs is the z score. That measures the node’s within-module Degree, relative to the average and normalised by the standard deviation (of within-module Degree, of all nodes in that module). The cut off originally assigned, in a cellular biology application, for classification as module hubs was *z* > 2.5 standard deviations above the average. In the present study some nodes are more than two standard deviations above the mean, so credibly could be classified as marginal hubs—thus they could be investigated further.

### 2.4 Link pathways

Having identified modules and key hubs and connector nodes analysis of their local link network can shed light of cortical organisation. To explore this the ideas implicit in temporal networks (Holme and Saramarki, 2012) were used to trace time evolving links to or from selected nodes. Assuming constant signal velocity, here taken to be 1 m/s, as an example typical of local circuits that may have unmyelinated axons, provides a scaling between link distances and time. Thus following links of longer pathlength also follows the time evolution of pathways. Link tracing covered all linked first nearest neighbours and extended to second linked neighbours, allowing for a 2 ms synaptic delay (i.e., equivalent to 2 mm extra pathlength). A Matlab code assembled all the links with a selected node[s], then resorted them into increasing link distances, or shells, of neighbours, and incrementally drew the evolving network of local links in 3D. This facilitated visualisation of link patterns around the key hubs. Thus locally clustered nodes and their local and global links readily became apparent.

All calculations were performed with Matlab codes available from standard repositories (BCT at, NIFTI tools at),^4^^,^^5^ or written Matlab codes lodged at https://github.com/BrainDynamicsUSYD/MarmosetCortex/.

## 3 Results

**FIGURE 3:**
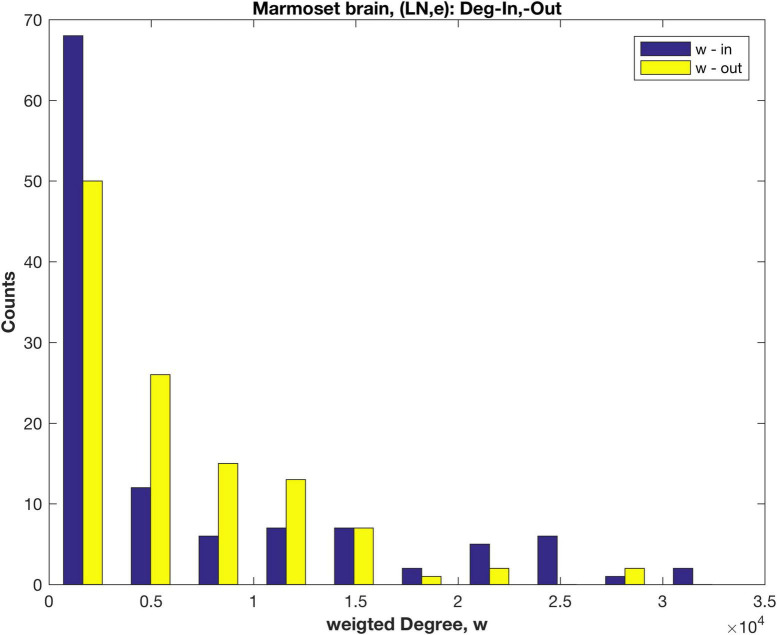
Distributions of the weighted in- and out- Degree, calculated using the rescaled measure LNe, for anatomical areas of the marmoset brain.

Here a direct link weight measure, LNe, is employed so that the underlying weight distribution for the in-links is revealed. LNe is the number of labelled neurons, extrinsic (i.e., not in the target area, and including all those detected in related source areas). The rescaled link weights, LNe are now in the range 0.03–10^4^ (this weight is dimensionless, being a cell count). The resulting link weight distribution (log_10_ scale, Supplementary Figure 2) can be compared with the distribution using the original fractional measure FLNe (Supplementary Figure 1). The weighted in- and out- Degree distributions are shown in Figure 3. Note that the un-weighted Degree (i.e., number of links. k) remains unchanged, and was shown in Figure 1.

Both the weighted in- and out-Degree distributions decay rapidly and in a similar fashion, in contrast to the results using FLNe (cf. Figure 2), where the in-Degree has a distinct pattern. Plots of a fitted exponential distribution (cf. Supplementary material 2.3 and Supplementary Figure 3) indicate that the weighted in-Degree falls off more rapidly than exponential, as also evident in Figure 3, compared to the weighted out-Degree. Possibly some low weight in-links are missing, since the in linkage data is incomplete with only 55 of the 116 anatomical areas being tracer injection sites. Here an extra 1611 links are included that these are out links from the non-sampled areas to any of the 55 injection sites. The present results are more in line with tracer results for Mouse (Oh et al., 2014; Pailthorpe, 2019) and EM based results for worm (Varshney et al., 2011) and mouse retina (Helmstaedter et al., 2013; Pailthorpe, 2016).

The log link weight–distance plot (Supplementary Figure 6) indicates an exponential decay with large scatter, as observed for other species, and a linear fit yields weight ∼ e^–dist/4.57^ (*R*^2^ = 0.16, with distance in mm). For comparison a log-log plot similarly displays large scatter, and produces a scaling law: weight ∼ dist^–2.00^ (*R*^2^ = 0.23), compared to weight ∼ dist^–2.05^ found for mouse (Rubinov et al., 2015). The difference between the two fits is too marginal to discriminate. For comparison, functional connectivity data in human brain produces a weight–distance decay midway between exponential and power law (Roberts et al., 2016).

### 3.1 Network modules and hubs, using LNe

The module analysis computed using marmoset FLNe is presented in Supplementary Table 2 and discussed in the Supplementary material 2.6. Module membership computed using the Infomap method with rescaled weights derived from LNe are summarised Table 2, with a full list attached at Supplementary Data. Probability flow is a key variable in the Infomap method and priorities nodes, and modules, based on the fraction of random walkers transiting a node—essentially it’s a proxy measure of local network traffic.

**TABLE 2:** Infomap modules for marmoset cortex ipsi-lateral links, using the rescaled LNe measure of link weights.

Module	Flow in module	Number of areas	Key areas	Lobes/Regions	Colour
1	0.250	12	A1-2, PF, S2e, AIP, PFG, A3a	Somato sensory, parietal	Yellow
2	0.150	11	A23c, A23b, A24d, A23a	Cing/RSP	Green—light
3	0.147	21	A11, A32V, A10, A9, A8b	pFC (Dl, M, Orb)	Red/pink
4	0.145	12	A8c, A8aV, A45, A47L	Motor, pFC (Dl, Vl, Orb)	Salmon
5	0.104	7	PG, MST, PE, V4T	Vis, parietal	Green—light
6	0.088	22	TPO, AuRT, AuPCB, AuCM	Aud, InsulCtx	Yellow/brown
7	0.073	14	TE3, V4, TEO, PGa-IPa, V3	TempCtx (V, L), Vis	Green–lime
8	0.044	9	V2, V3a, PEC, A19DI	Vis, parietal	Salmon
Orphans	0.0	8	A29a-c, A29d	Cing/RSP	Grey

Key members are listed in order of probability flow; colour coding follows the Marmoset Atlas and is used in the figures.

Here the module membership is somewhat similar to that found with the original FLNe measure (cf. Supplementary Table 2), however, the order is changed, both within and between modules. Now the somatosensory system hosts the most network traffic, and here is separated from the motor system. The modules containing the largest number of anatomical areas are #3, associated with pFC and #6, auditory cortex. Note that this module assignment likely will be revised when in-links to the remaining 61 (i.e., 116–55) targets are measured and reported. Here also the auditory system constitutes its own module, and the previous modules #6 and #7 (cf. Supplementary Table 2) are merged into a single module (6). The visual system is present across three modules. The contrast between Table 2 and Supplementary Table 2 is that differences in the weight of in-links are accounted for, rather than being factored out as in the common normalisation forced by the fractional measure, FLNe.

For comparison a simple implementation of the Louvain method (Blondel et al., 2008), an agglomerative method, was also used to find the modular decomposition of the marmoset cortex, generating 7 modules that are closely aligned with the InfoMap results reported here. The modularity metric is *Q* = 0.52 (Newman and Girvan, 2004). It reported no singletons (isolated nodes), in contrast to the InfoMap method. Interestingly the Louvain method when applied to an unweighted adjacency matrix did not decompose the network into modules, indicating that the link weights play a critical role in resolving the component modules. A separate analysis of the full connected 55 x 55 connectivity matrix using FLNe and a more comprehensive Louvain-like method (Liu et al., 2020), with tuned sensitivity and consensus sampling, produced 4, 5, or 8 modules and assigned prominence to frontal and auditory areas, in agreement with the present analysis.

**FIGURE 4:**
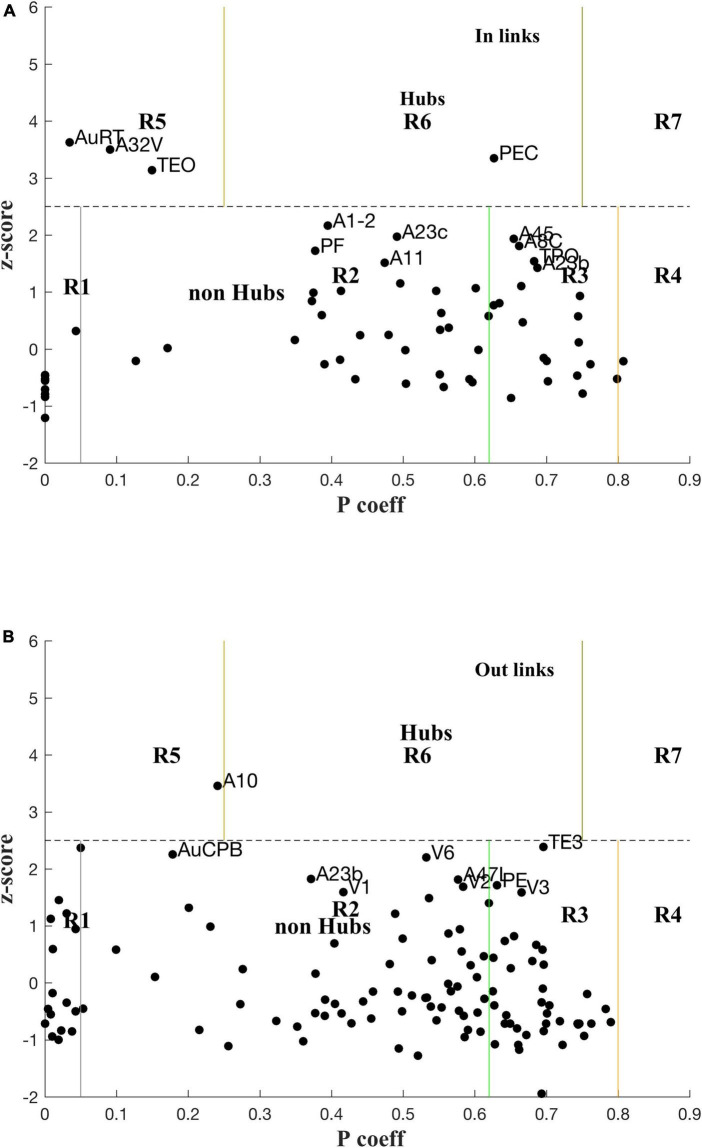
Plot of z-scores vs. participation coefficients for the marmoset cortex, calculated using the LNe data (cf. Methods 2.3, Supplementary material 2.3) for in-links **(A)** and out-links **(B)**.

Classification of nodes into types of hubs and connectors is illustrated in Figure 4 with plots of z_i_ vs. P_i_, the module membership score (within-module Degree) and participation coefficient, respectively, of each node (cf. Methods 2.3), for both in- and out-links. The zones for each class of role are labelled. Hubs and nearby, possible candidates are also labelled. The boundaries were based on heuristics developed for cellular metabolic networks (Guimerà and Nunes Amaral, 2005), e.g., *z* > 2.5 for hubs, and that may need to be revisited for cortical networks.

The rescaled link weights preserve differences of in-links between various nodes, and now reveals in-hubs: PEC (Parietal) as a Connector in-hub (R6), and AuRT (Aud), A32V (M-pFC), and TEO (Temporal) as Provincial in-hubs (R5). These in-hubs are missed in the analysis using FLNe (cf. Supplementary Figure 7). Three nodes, A1-2, A23c, and A45, are close to the borderline for in-hubs, so might be marginally classified as hubs since the z-score cutoff (2.5) is somewhat arbitrary in that it was originally determined by a heuristic rule for another biological application (Guimerà and Nunes Amaral, 2005). There are no out-link Connector Hubs (R6; cf. Figure 2), but PGa-IPa might be considered as marginal; A10 is a Provincial out-hub (R5), with AuA1 and AuCPB as marginally so. TE3 is a borderline connector out-hub (R6). The full list of P, z values for the 116 nodes are attached at Supplementary Data.

For in-links, four nodes are prominent non-hub connector nodes (R3; i.e., well linked to other modules): A45, A8c, TPO, and A23b; another 13 are also R3 but less well linked: PG, V4, TE3, A47L, PGa-IPa, A23a, V4T, AIP, PFG, A8aD, A6M, LIP, and V5. Note that there are commonalities with the lists using FLNe (Supplementary material), but also differences. For out-links six nodes are prominent non-hub connector nodes (R3): PE, V3, A8aV, PG, V4, and TPO–again in order of decreasing P; another 22 nodes are classified as R3, but are less well connected. One node for which in-links were measured (i.e., an injection site) is classified as peripheral (R1) for in-links: A32V, along with eight nodes that were not measured for in-links: AuAL, AuR, A25, AuRTL, AuRM, TPro, AuRTM, and APir. As a check these network based classifications need to make sense biologically.

**FIGURE 5:**
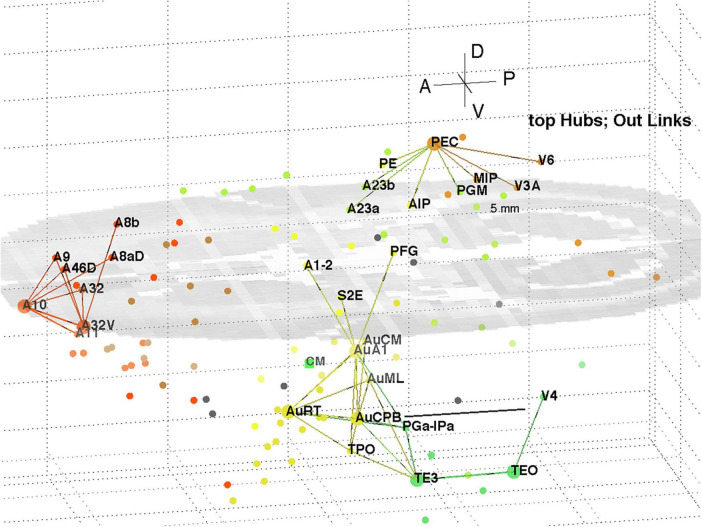
3D plot of network hubs and their out-links of length <5 mm. Nodes are coloured by their module membership (cf. Table 2) and links by the source node membership. The mid-level horizontal plane image is a slice from the marmoset cortex volume image to aid perspective, along with the background grid. Ipsi-lateral links for the left hemisphere were reported. The background grid is 5 mm × 2 mm and the A-P scale bar (near V4) is 5 mm.

**FIGURE 6:**
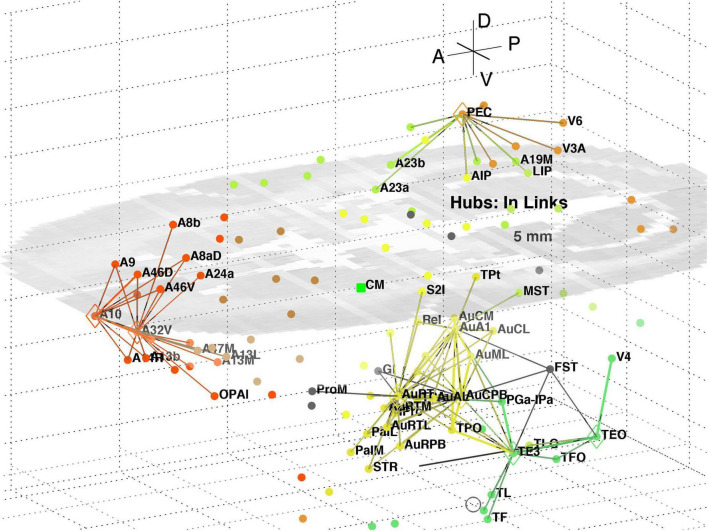
3D plot of network hubs and their in-links of length <5 mm. Nodes are coloured by their module membership (cf. Table 2) and links by the target node membership. Other details as in Figure 5.

The top 8 hub nodes are: A10, TE3, AuA1, AuCPB, AuRT, A32V, TEO, and PEC. They are plotted in 3D with their -out and in-links in Figures 5, 6, respectively. Only links of weight >1 and length <5 mm are shown for clarity, and to reveal the local clustering. The very weak 135 out- and 51 in- links, of weight <1, are omitted to avoid clutter. Colours follow the marmoset Atlas (Paxinos et al., 2012); a grey underlay is introduced to provide some contrast, which may shift the colours. All nodes are plotted but labels and links are not drawn if the latter are longer than 5 mm for clarity.

**FIGURE 7:**
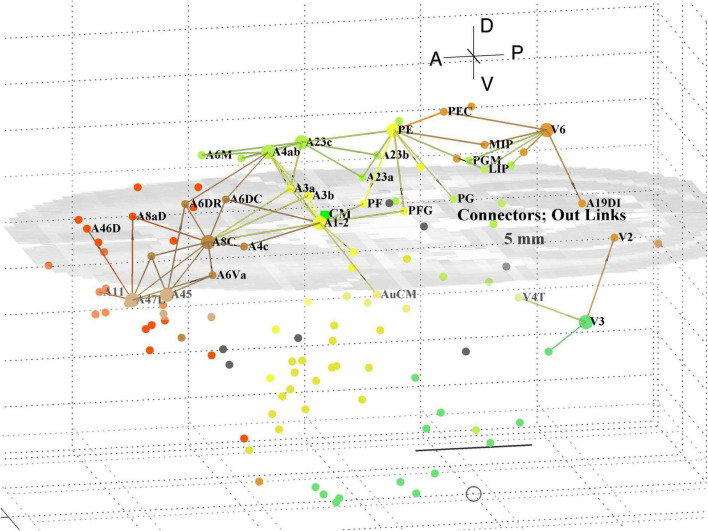
3D plot of network connector nodes and their iout-links of length <5 mm. Nodes are coloured by their module membership (cf. Table 2) and links by the source node membership. Other details as in Figure 5.

**FIGURE 8:**
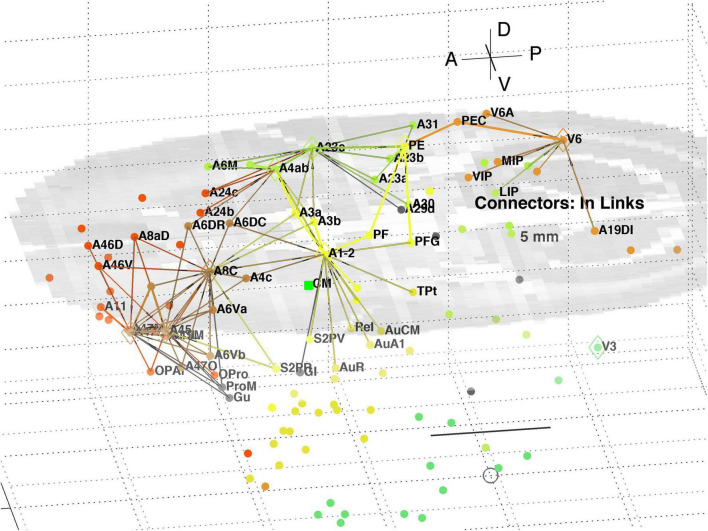
3D plot of network connector nodes and their in-links of length <5 mm. Nodes are coloured by their module membership (cf. Table 2) and links by the target node membership. Other details as in Figure 5.

A separate analysis of the 55 x 55 sub-network using participation coefficients only (Liu et al., 2020) identifies frontal, auditory and association areas as most hub like, followed by visual areas, and singles out A10 as having unusually large out strength, in agreement with the present study. Continuing the path tracing of Figures 5, 6 to longer distances (∼ 10 mm) reveals direct links from the auditory areas AuCPB and AuA1 to the frontal areas; and bridging nodes between the visual and auditory sensory areas to be PEC, TEO and TE3; with PEC later on linking to motor areas. Similarly, the top 9 connector nodes are: PE, V3, A47L, V6, A4ab, A45, A1-2, and A23c. They are plotted in 3D, along with their close out- and in-links in Figures 7, 8, respectively. These show the evolving fabric of links between the hub clusters.

Together these visualisations show the major local clusters and their interlinkages in marmoset cortex. Sensory Pathways were traced (cf. Methods 2.4) from visual and auditory cortex to anterior cortical areas. Aside from relatively weak direct links (e.g., V2–A8aV, V2–A32) high weight links to the frontal areas from visual areas requires two steps, via intermediate transit nodes: e.g., V2 via V4, V5, MST, TEO, TPO, and Opt. Those variously on-linked to areas of the pFC cluster: A32, A46D, A10, A11, A32V, and A9. For the auditory areas direct links are from AuA1 via TPO to the hubs A32V and A11, and from AuCPB direct to A10, A32V, A11, and A46D, all targets in the pFC cluster.

### 3.2 High traffic links derived from LNe

**FIGURE 9:**
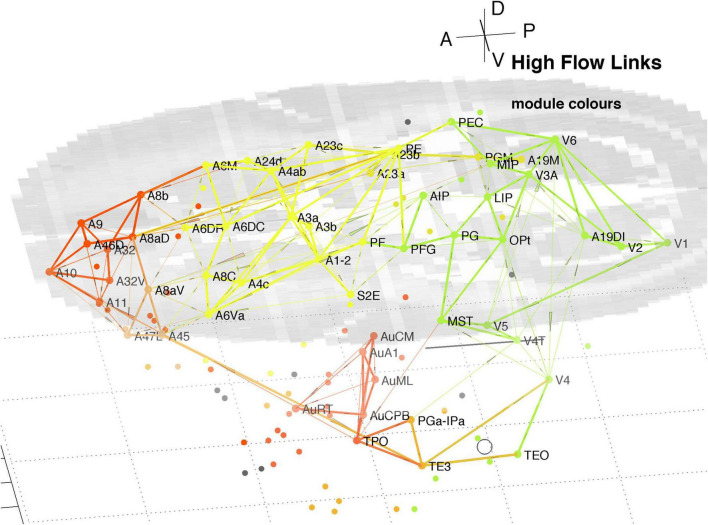
3D plot of all nodes and the top 15% highest probability flow links in the marmoset cortex. Higher traffic links are in bold. Colours identify module membership (cf. Table 2) and follow the marmoset Atlas. The mid-level horizontal plane image is a slice from the marmoset cortex volume image to aid perspective, along with the background grid. The A-P scale bar is 5 mm.

The InfoMap analysis also yields the probability flow through individual nodes which, in turn, allows calculation of flow over links. This flow is the fraction of random walkers that pass over the node or link. That allows identification of the highest signal traffic links in the whole cortex, as displayed in Figure 9. It displays the top 200 of both in and out links, being the top 15% highest probability flow amongst all cortical links. Those linkages follow a similar pattern to that derived via hubs and connectors as shown in Figures 5–8. The colours identify module membership (cf. Table 2) and follow those of the marmoset Atlas. The mid-level horizontal plane image is a slice from the marmoset cortex volume image (Paxinos et al., 2012; see text footnote 1) and aids perspective in the 3D view.

The clustering evident in Figure 9 reiterates that found above around hubs and connector nodes and highlights a densely linked cluster in pFC. The organisational picture shown in Figure 9 is consistent with the finding of key nodes, high degree, that are locally densely connected and well-connected between the different regions found in the previous analysis of the 55 node sub-network (Liu et al., 2022).

It is reassuring that methods based on node participation and on link traffic yield similar results. While not conclusive Figure 9 suggests parallel pathways from sensory areas: from auditory cortex direct to multiple areas of pFC; and from visual cortex via parietal areas to motor areas and pFC. There is some cross coupling of V4 via temporal areas, and of V1, V5, and V6 via MST to auditory cortex. The visual pathways are consistent with a detailed review of the marmoset visual system in Solomon and Rosa (2014), which noted MST as a transit hub from V6.

## 4 Conclusion

A rescaling of the original marmoset structural connectivity data reveals the underlying in-link weights and facilitates a more complete network analysis of the cortical network. The in- and out-link weights then follow similar distributions (Figure 3), with rescaled link weights in the range 0.03–10^4^. The weight measure is the number of labelled neurons in a target area due to links from a source area, in keeping with the mouse brain data (Oh et al., 2014; Pailthorpe, 2019), but in contrast to the EM measurements of synaptic contacts or areas in mouse retina (Helmstaedter et al., 2013; Pailthorpe, 2016) and fly (Chklovskii et al., 2010). While there are a number of options, the relationships between LN and injection and target volumes (Supplementary Figures 4, 5) suggest that LNe is a viable measure of link weight. The scaling factor to convert labelled neuron counts to synaptic weights is not clearly defined and warrants further study, thus the interpretation of weight <1 is yet to be clarified.

The distributions of in- and out-weighted Degree in Figure 3 suggest that some in- links of low to medium weight are missing. A similar plot for mouse brain (Pailthorpe, 2019) shows both distributions being quite similar–again suggesting some missing in-weight data here. This is consistent with sampling only 55 source nodes, being the 55, of the 116, anatomical areas used as tracer injection sites. In due course such data should be available and complete the picture emerging here. The link weight—distance relationship, based on the scattered data, marginally follows both an exponential decay, or a power law decay (Supplementary Figure 6) consistent with other species.

A decomposition of the network using InfoMap produces 8 modules (Table 2) aligned with dominant cortical regions, and enables the subsequent analysis. Classification of within and between module linkage patterns, using z-scores and participation coefficients, identifies key hubs and connector nodes (Figure 2). This can be applied to both in and out links. Hubs, with many links between modules or many within a module, provide an anchor for local, densely interlinked clusters (Figures 5, 6) in pFC, association, auditory and visual areas. By contrast, connector nodes have many links to other modules and are waypoints between the clusters (Figures 7, 8). Those linkage patterns are explored by following evolving link formation at increasing distances, which reveals the gradual shift from local to global linkage patterns. The analysis reveals PEC, AuRT, A32V, and TEO as key in-hubs, with A45, A8C, A1-2, A23c, PF, and A11 as possible candidates. A10 is the dominant out-hub, with TE3, AuCPB and AuA1 as candidates also worthy of further investigation.

A separate, extensive network analysis of the fully connected marmoset sub-network of 55 nodes (55 x 55 linkage data) (Theodoni et al., 2022) using the fractional weight measure FLNe produced a hierarchical ordering of areas, from frontal to visual areas. At the top was A6 (motor); AuCPB (auditory); A32, A8aD (frontal cortex); A4c, A6M (motor); and later A11, A46aD, A10 (frontal cortex); continuing through association and somatosensory areas, and on to visual areas at the lowest level. That is somewhat consistent with the ordering in the top 4 modules identified in the present study. The analysis by Liu et al. (2022) identifies frontal and auditory cortex as prominent, separate modules, consistent with the present study.

Another analysis, of cat and macaque hub nodes (Sporns et al., 2007), used centrality measures and the participation coefficient only, but not the z-score. It was applied to unweighted links and to motifs, which generally are small clusters, typically of 3 nodes. That different methodology identified V4 as a key hub, somewhat consistent with the present study, and A46 (DlpFC) as another key hub. The inclusion of link weights and the z-P classification, used herein, produced hubs in pFC and in sensory, association and motor areas. Functional connectivity in marmoset brain, studied by resting state fMRI (Belcher et al., 2016). measured as local Functional Connectivity Density, identified 11 hubs across the whole brain. In the cortex: area A24a (Cingulate) was the most prominent, followed by V6, A19M (vis.), A23a,b (posterior Cingulate), and at lowest strength: V1 and V2.

Separately high traffic links across the cortex (Figure 9) confirm the overall picture presented herein and illustrates parallel auditory and visual pathways. Some features of those paths are consistent with a detailed review of the visual system in marmoset (Solomon and Rosa, 2014). The most significant cluster in pFC comprises 6–8 nodes, two of which (A10, A32V) are dominant hubs with a third (A11) nearly so. This suggests a central role in the cortical network and warrants further investigation.

The key limitation of this study is that it used incomplete linkage data, with only 55 of 116 areas sampled by tracer injection. One can reasonably expect that those sites were chosen as the most biologically important, so the present analysis produces a partial picture of significant network structures. It extends prior analyses of the marmoset data by including some 1611 links that are missed by trimming the data to 55 x 55 sources and targets. That excluded 26% of the total link weight measured which now is included in the present study. With the eventual availability of more data that emerging picture can be completed. A separate tracer study of marmoset cortical links (Watakabe et al., 2023) provides information at columnar level, in a parcellation free analysis, and needs to be integrated with these area level studies. Another conceptual framework is provided by the structural model (García-Cabezas et al., 2019) that is based on cortical types rather than parcellation into areas. It allows a complimentary approach to analysing data, such as link weight-distance relationships (Aparicio-Rodríguez and García-Cabezas, 2023), that can augment network-based studies. Analysis using such new data and biological frameworks will enhance the multiple studies discussed herein.

## Data availability statement

The original contributions presented in the study are included in the article/Supplementary material, further inquiries can be directed to the corresponding author.

## Author contributions

BP: Conceptualization, Data curation, Formal analysis, Investigation, Methodology, Software, Validation, Visualization, Writing – original draft, Writing – review and editing.

## Footnotes

1
http://marmosetbrain.org

2
www.mapequation.org

3
https://sites.google.com/site/findcommunities/

4
https://sites.google.com/site/bctnet/

5
http://mathworks.com/matlabcentral/fileexchange/8797
